# Characterization of hepatitis B virus in Amerindian children and mothers from Amazonas State, Colombia

**DOI:** 10.1371/journal.pone.0181643

**Published:** 2017-10-10

**Authors:** Carlos Mario Jaramillo, Fernando de La Hoz, Alexandra Porras, Diana di Filippo, Luz Angela Choconta-Piraquive, Edra Payares, Neyla Montes, Maria-Cristina Navas

**Affiliations:** 1 Grupo de Gastrohepatologia, Facultad de Medicina, Universidad de Antioquia, UdeA, Medellin, Colombia; 2 Grupo de Epidemiologia y Evaluación en Salud Pública, Universidad Nacional, Bogota, Colombia; 3 Laboratorio Departamental de Salud Publica del Amazonas, Leticia, Colombia; 4 Coordinacion Salud Publica, Alcaldia de Puerto Nariño, Puerto Nariño, Colombia; CEA, FRANCE

## Abstract

**Background:**

Hepatitis B Virus (HBV) infection is a worldwide public health problem. In the 1980’s a highly effective and safe vaccine against HBV was developed, although breakthrough infection still occasionally occurs because of the emergence of escape mutants. The aim of this study was to identify HBV genotypes and escape mutants in children and their mothers in Amerindian communities of the Amazonas State, Southern Colombia.

**Methods:**

Blood specimens collected from children and mothers belonging to 37 Amerindian communities in Amazonas state, were screened for HBsAg and anti-HBc using ELISA. The partial region containing the S ORF was amplified by nested PCR, and amplicons were sequenced. The phylogenetic analysis was performed using the MEGA 5.05 software.

**Results:**

Forty-six children (46/1275, 3.6%) and one hundred and seventy-seven mothers (177/572, 30.9%) were tested positive for the anti-HBc serological marker. Among them, 190 samples were tested for viral genome detection; 8.3% (2/31) serum samples obtained from children and 3.1% (5/159) from mothers were positive for the ORF S PCR. The predominant HBV genotype in the study population was F, subgenotype F1b; in addition, subgenotype F1a and genotype A were also characterized. Two HBV escape mutants were identified, G145R, reported worldwide, and W156*; this stop codon was identified in a child with occult HBV infection. Other mutations were found, L109R and G130E, located in critical positions of the HBsAg sequence.

**Conclusions:**

This study aimed to characterize the HBV genotype F, subgenotypes F1b and F1a, and genotype A in Amerindian communities and for the first time escape mutants in Colombia. Further investigations are necessary to elucidate the frequency and the epidemiological impact of the escape mutants in the country.

## Introduction

Hepatitis B virus (HBV) infection is a worldwide public health problem. It is estimated that around 2 billion people have been infected and that more than 240 million are chronically infected [1]. Colombia is a country of low endemicity for HBV infection considering the average incidence of hepatitis B registered from 2008 to 2015 (3.11 to 4.68/100.000) [2,3,4,5,6,7,8]. However, the report by state showed a high heterogeneity, such as the Amazonas State with an incidence of 18.3 per 100.000 inhabitants in 2015, and where more than 50% of the adults have been infected at some point with HBV [7,8].

HBV is a partially double-stranded DNA virus that belongs to the family *Hepadnaviridae*. The viral genome has four overlapping open reading frames (ORFs): ORF S, P, C and X. ORF S encodes the three forms of the surface antigen (HBsAg) L, M and S [9]. All three HBsAg contain a region (amino acids 100–164) known as the antigenic loop polypeptide which include the "a" determinant (amino acids 124–147) (9–12).

A recombinant vaccine against HBV was developed in the 1980’s. This vaccine has an efficiency of 95% and has been implemented in 92% of countries around the globe [10,11]. The HBV vaccination program was implemented for the first time in Colombia in 1993 targeting newborns and children < 5 years using a 0-1-2 scheme with a monovalent hepatitis B vaccine. Since 2001 the vaccination schedule was modified to one monovalent birth dose and three doses of a pentavalent vaccine (diphteria, tetanus, pertussis, *Haemophilus influenzae* type b and hepatitis B). A decrease in prevalence of HBV infection after the implementation of the vaccine was described in highly endemic regions in Colombia, such as Amazonas state [12,13].

The recombinant vaccine contains HBsAg, a protein that is highly conserved in all HBV genotypes, in spite of the high mutation rate of HBV due to its highly error-prone viral polymerase. The low variability of this region is due to the pivotal role as infectivity determinant of HBV [14].

However, mutations in the “a” determinant can arise under pressure of immune response in infected or vaccinated individuals or after immunoglobulin prophylaxis therapy [15,16,17]. Monitoring these mutations in vaccinated populations should be part of the evaluation of HBV vaccination programs.

A serological survey of HBV infection and carriage was conducted in several rural areas of the Amazonas State, in order to monitor the HBV infection control after 18 years of vaccination. This report presents the genotyping analysis and identification of escape mutants, on a sample of Amerindian children and mothers found to be positive for Anti-HBc in the main study.

## Methods

### Study population

A population-based cross-sectional survey designed to evaluate the prevalence of HBV infection and vaccine coverage, was carried out among rural communities belonging to two municipalities, Puerto Nariño and Leticia, and one village, Tarapaca, in the Amazonas State (Southern Colombia). Moreover, the study aimed to characterize the viral genotypes and to identify HBV escape mutants.

The study was carried out between June 2010 and June 2011 in 37 Amerindian communities settled on the banks of the Putumayo and Amazon rivers and their tributaries (Cothue, Amacayacu, Loretoyacu, Atacuari and Boyahuazu).

For the main study, the sample size was calculated to estimate a prevalence of at least 1% of HBsAg positives among children. It showed that 1000 children would be enough to estimate that parameter within a confidence limit of 0.5% with 95% confidence level. Overall, the study population included 1275 children (6 months to 11 years old) and 572 mothers.

Villages were selected for the study based on previous work that identified them as having a high prevalence of HBV infection [12]. In the villages selected for the study, every household was visited and the parents invited to participate in the study. All eligible children found in any given household were included in the study if their caretakers approved to participate. Households were visited starting from the farthest and going to the nearest to reach the center of the community.

The mother or the grandmother of the children signed informed consent; a questionnaire containing demographic data was filled and the vaccination history of every child was recorded using information from the vaccination card. All vaccination data of each child was confirmed. The vaccination data of 7% of children was not available.

The status of vaccination against hepatitis B was defined using the following criteria: complete vaccination: child older than 6 months who had the birth dose and three additional doses regardless of the time. Incomplete vaccination: child older than 6 months who had the birth dose and / or any of the three subsequent doses, 2, 4 and 6 months. Timely vaccination against hepatitis B: child older than 6 months who received the newborn dose within 48 h of birth and the three subsequent doses at 2, 4 and 6 months.

The sources of information were the vaccination card of each one of the children and the database of Amazonas and Leticia health system.

### Specimen collection and testing

A blood sample was taken from both children and mothers. However, 23 mothers’ blood samples were not obtained because they were out of the community during the visit and therefore were not included in the analysis.

The serum was obtained and stored at -4°C until the time of transport to the Public Health Laboratory in Leticia where the samples were kept at -70°C. All samples were evaluated for the serological markers anti-HBc and HBsAg using commercial ELISA kits (UMELISA anti-HBc and UMELISA AgHBs PLUS, TecnoSuma, Cuba). Anti-HBc negative samples were analyzed for the anti-HBs marker using a commercial ELISA kit (UMELISA anti-AgHBs, TecnoSuma).

Anti-HBc+ samples (31 from children and 159 from mothers) were sent to the laboratory of the Universidad de Antioquia for analysis by PCR of the HBV genome detection and sequencing. The other anti-HBc+ samples were not available for this analysis.

DNA extraction was performed from 200 μL of serum sample using a commercial kit (QIAmp DNA minikit, QIAGEN, Germany); some modifications of centrifugation times and additional washings were included. An HBV+ serum and a liver tissue sample from a bank of explant tissues were used as positive controls in the PCR.

Three PCRs were performed to amplify the three regions of the HBV ORF S (S, PreS1 and PreS2), using primers previously described [18] or designed for the present study (P3006f/P213r nucleotide position 3006–3028 5’-GCCGCGTCGCAGAAGATCTCAA-3’ and 213–192 5’-AAACACACCGCCTGTAACACGA-3’). The S region was amplified using a nested PCR with 5 units of Biolasa polymerase (Bioline, UK), 2.5 mM of each dNTPs, 10x reaction buffer, 25mM of MgCl_2_, 10 μM of YS1 and YS2 primers and 5 μL of DNA in a final volume of 50 μL for the first round. For the second round, 2 μl of first round PCR product was added to 23 μl of the reaction mixture in the same conditions as the first round using primers S3s/S3as [19].

Amplification of region PreS1 was carried out with P1/P2 primers for the first round and 2440p/58n primers for the second round, in a final volume of 50 μL and 25 μL, respectively. The PreS2 region was amplified using the P3006f/P213r primers in a final volume of 25 μL for both rounds.

The PCRs products were visualized in 2% agarose gels stained with SYBR green at a 10μg/mL concentration; the gels were photo-documented with the equipment of the 2UV Transilluminator Digital Imaging System (UVP, USA).

#### DNA sequencing

Sequencing was performed on an automated BigDyeTM terminator (Macrogen Korea). Sequence edition was performed with the Vector NTI (Invitrogen) software, the Seqman DNAstar software and BioEdit Package Version 7.0.9. For this analysis, a prototype sequence of HBV genotype F was used (accession number HE974369).

#### Sequence analysis and escape mutants

The analysis of escape mutants was performed using the MEGA 5.05 software, and using as reference an HBV isolate from a Colombian patient (accession number FJ- 589070) reported by Cortes *et al*. After nucleotide alignment, the most probable sequence of amino acids for ORF S was determined and escape mutants and polymorphisms in the “a” determinant were identified.

#### Phylogenetic analysis

The phylogenetic analysis was carried out using the Neighbor-Joining method in the MEGA5.05 software. Evolutionary distances were calculated using the Kimura 2-parameter method + Gamma distribution, and the bootstrap consensus tree inferred from 1000 replicates. The reported sequences in GenBank of all HBV genotypes and subgenotypes of genotype F, of different countries and year of publication were included in the analysis. Nucleotide sequence data have been deposited into the GenBank database under the accession numbers MF400836-MF400842.

### Ethics statement

The project was approved by the ethic committee of the Universidad Nacional de Colombia and by the public health authorities of the Amazonas State. Moreover, the community approval was obtained after explaining the project to the leaders of Amerindian communities Aticoya (Asociacion Ticuna, Cocama y Yagua) and Acitam (Asociacion de Cabildos Indigenas del Trapecio Amazonico).

## Results

### Study population

The study was carried out on 1275 children and 572 mothers from 37 Amerindian populations belonging to Puerto Nariño (17 communities), Leticia (12 communities), and Tarapaca (8 communities) in Amazonas State. Most children and mothers belonged to the Ticuna ethnic group (95.4%) and the others to the Cocama, Huitoto, Yagua, Bora, Inga, Ocaina, Ignano, Yucun and Miraña ethnic groups.

Among the children, 531 samples were collected in Puerto Nariño, 449 in Leticia and 295 in Taparaca. Among the mothers, 245 were from Puerto Nariño, 199 from Leticia and 128 from Tarapaca.

The mothers average age was 32 ±8.3 years with a range between 16 and 59 years while the children’s average age was 5 ±3 years with a range between 6 months and 11 years.

From the child study population, the vaccination card was available in 93% of the cases. Coverage of HBV vaccine was 81% for the monovalent birth dose; however, timely vaccination only occurred in 34.1% of the cases. Coverage was 89.7% for the first dose of pentavalent vaccine, 89.7% for the second dose and 86.9% for the third dose. Taking in account all the data, hepatitis B coverage was 90.8% of the child population, although only 22.8% were timely vaccinated (monovalent dose within 48 h after birth and pentavalent doses given at 2, 4 and 6 months).

### Serological markers and viral genome detection

A total of 46/1275 (3.6%) samples obtained from children were positive for anti-HBc marker, and among them 7/1275 (0.5%) were positive for both markers anti-HBc and HBsAg. In addition, 276 samples that were anti-HBc negative in the children were tested for anti-HBs; 21.3% (59/276) of samples were reactive. Children with timely vaccination (birth dose, and doses at 2, 4 6 months) had 70% less risk of being HBV infected compared to those who were not (OR = 0.23, 95% CI 0.09–0.51). On the other hand, being born to mother HBsAg+ increased 2.5 fold the risk of being HBsAg carrier (OR = 2.45, 95% CI 1.33–4.46).

Thirty-one anti-HBc+ samples (24 samples anti-HBc +/HBsAg–and 7 anti-HBc +/HBsAg +) were analyzed for the HBV genome. Two samples (2/31, 8.3%) were positive for the ORF S PCR.

The serological marker anti-HBc was detected in 176/572 (30.9%, (CI 95% 27.1–34.7) serum samples obtained from mothers and among them 52/572 (9%, CI95% 6.4–11.1) were positive for both markers anti-HBc and HBsAg. Five anti-HBc+ serum samples were positive for the ORF S PCR (5/159, 3.1%) out of 159 samples analyzed (110 samples anti-HBc +/HBsAg–, and 49 anti-HBc +/HBsAg +) (Table 1). All the positive blood samples were analyzed in duplicate.

**Table 1:** HBV infection markers in Amerindian children and mothers from the Amazonas State.

Serological and molecular markers of HBV infection	Children n = 1275	Mothers n = 572
anti-HBc+	46/1275 (3.6%)	177/572 (30.9%)
anti-HBc + / HBsAg +	7/1275 (0.5%)	52/572 (9%)
anti-HBc + / HBsAg–	38/1275 (2.9%)	124/572 (21.6%)
Viral Genome detection (S region)	2/31 (6.4%)	5/159 (3.1%)
anti-HBc + / HBsAg—/ DNA HBV +	2/24 (8.3%)	0/110 (0%)

The low number of samples successfully amplified could be due to the detection limit of the PCR strategies and the low viral load present in samples taking in account that all were obtained from asymptomatic individuals and that most samples are negative for the HBsAg marker. Moreover, the conditions of the samples storage and shipping during the fieldwork could have also impaired the detection of the viral genome.

The 7 samples positive for the S region (2 from children and 5 from mothers) were analyzed for the PreS1 and PreS2 regions. Amplifications of the PreS1 and PreS2 regions were obtained in samples 045_SJAtacuari_AM and 051_Pto Esperanza_AM (Accession number MF400836 and MF400838, respectively), both obtained from mothers.

### Identification of escape mutants in the ORF S of the HBV genome

Four mutants were identified in three serum samples from Amerindians. The first escape mutant had change from guanine to adenine in nucleotide 587, resulting in the amino acid change from glycine to arginine in residue 145 (G145R) of the HBsAg; this mutation was present in sample obtained from a mother (127_Boyahuazu_AM Accession number MF400839). The second escape mutant identified had a change from guanine to adenine in nucleotide 621, which generated a stop codon in position 156 (W156*); this mutation was identified in sample 189_Tarapaca_AM (Accession number MF400842) obtained from a child with Occult HBV infection (OBI) (Table 2).

**Table 2:** Demographic data and markers of HBV infection in Amerindian children and mothers from the Amazonas State.

Sample	Ethnic Group	Community	Age (years)	Anti-HBc	HBsAg	Mutation ORF S	Genotype
020_SJ Atacuari_AM	Ticuna	San Juan de Atacuari	49	+	+	L109R G130E	F1a
045_SJ Atacuari_AM	Yagua	San Juan de Atacuari	33	+	+		F1b
048_ Naranjales_AM	Ticuna	Naranjales	23	+	+		F1b
051_Pto Esperanza_AM	Ticuna	Puerto Esperanza	28	+	+		F1b
127_Boyahuazu_AM	Yagua	Boyahuazu	32	+	+	G145R	F1b
180_Pto Esperanza_AM	Ticuna	Puerto Esperanza	4	+	-		F1b
189_Tarapaca_AM	Cocama	Tarapaca	3	+	-	W156*	A

**Fig 1:**
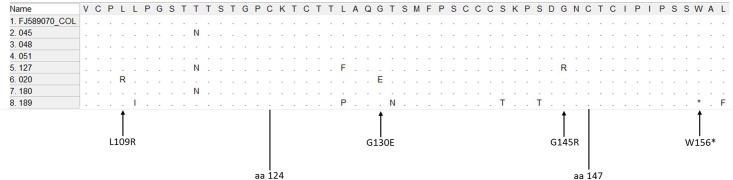
Alignment of amino acids corresponding to the major hydrophilic region (MHR) sequence of HBsAg of HBV, using the MEGA 5.05 software. The samples 020_SJ Atacuari_AM, 045_SJ Atacuari_AM, 048_Naranjales_AM, 051_Pto Esperanza_AM, 127_Boyahuazi_AM, 180_Pto Esperanza_AM and 189_Tarapacá_AM, were aligned with the reference sequence FJ589070.1 from GenBank. Arrow: identified escape mutants and potential escape mutants, amino acids 124–147: “*a*” determinant.

Furthermore, two potential escape mutants were found in a sample obtained from a mother (020_SJ Atacuari_AM Accession number MF400840). One had a thymine to guanine mutation in nucleotide 480, resulting in arginine instead of leucine in residue 109 (L109R) of HBsAg. The second mutation, guanine to adenine in nucleotide 543 resulted in the change of glycine to glutamic acid in position 130 (G130E) of the HBsAg (Fig 1 and Table 2).

Interestingly, none of the children born to the positive hepatitis B mothers was positive for the anti-HBc marker and none of the mothers of the two children positive for HBV infection was positive for the HBsAg marker (Table 3).

**Table 3:** HBV serological profile of mother/child pair of the study population.

Sample	HBV serological markers of Mother	HBV serological markers of children
Mother 020_SJ Atacuari_AM	Anti-HBc +/ HBsAg +	None of her 6 children was anti-HBc +
Mother 045_SJ Atacuari_AM	Anti-HBc +/ HBsAg +	None of her 3 children was anti-HBc +
Mother 048_Naranjales_AM	Anti-HBc +/ HBsAg +	None of her 2 children was anti-HBc +
Mother 051_Pto Esperanza_AM	Anti-HBc +/ HBsAg +	None of her 3 children was anti-HBc +
Mother 127_Boyahuazu_AM	Anti-HBc +/ HBsAg +	None of her 4 children was anti-HBc +
Child 180_Pto Esperanza_AM	Anti-HBc +/ HBsAg -	Anti-HBc +/ HBsAg -
Child 189_Pto Tarapaca_AM	Anti-HBc -	Anti-HBc +/ HBsAg -

### Genotyping of HBV by sequencing

**Fig 2:**
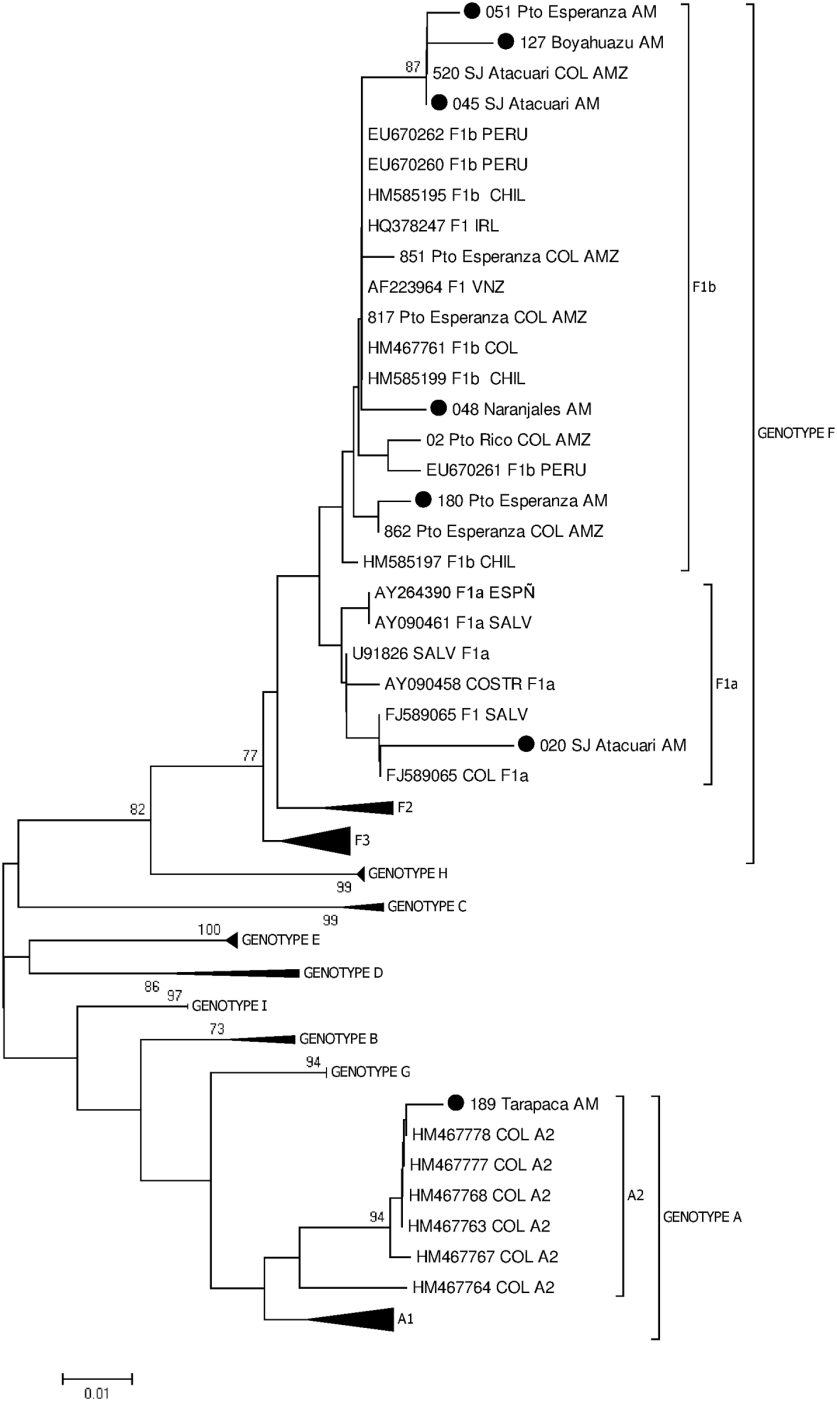
Phylogenetic tree without root of the S ORF (422–758 nt) of the HBV genome. The study sequences shown with red plots belong to the vaccinated children and the blue plots belong to the mothers. The sequences were compared with sequences of HBV genotypes A-I. The tree was generated using the MEGA 5.05 software, method Neighbor-Joining, model Kimura 2 parameters plus distribution gamma; the bootstraps were obtained with 1000 replicas.

Genotype analysis was carried out using a sequence of 336 nt (422–758 nt) of the S region. Ninety-four sequences of HBV genotypes A-I were obtained from GenBank; the phylogenetic analysis of these sequences generated the expected clusters. The branches were supported with bootstrap values >70 for each genotype (Fig 2).

The phylogenetic analysis showed that 6 sequences belonged to genotype F, 5 were located in the cluster of the subgenotype F1b, and 1 in the cluster of the subgenotype F1a. The remaining sequence belonged to the cluster of genotype A (Table 2).

## Discussion

Even the average incidence of hepatitis B in Colombia corresponds to low endemicity, there are several high prevalence regions in the country well described such as Amazonia, Orinoquia, the Sierra Nevada de Santa Marta and the Serrania de Perija. The heterogeneity of HBV prevalence in the country is mainly due to ethnic, socioeconomic, cultural and geographic differences [1,20,21,22,23]. Furthermore, recently a low coverage of timely birth dose of the vaccine in rural communities of the Amazonas state was demonstrated, in spite of the coverage birth dose of hepatitis B vaccine reported in Colombia (>80%). Indeed, the analysis of 938 children of the main study showed that only 37% corresponded to a timely birth dose and the average time for monovalent dose administration was 14 days after delivery. This analysis demonstrated the difficulties encountered in eliminating perinatal transmission in rural areas of Colombia, in particular in Amazonas State and the importance there is to improve the conditions for a timely birth dose in the rural population and increasing the number of newborns delivered in health facilities [24].

Despite the low coverage of timely birth dose, the efficiency of the hepatitis B vaccination program in Colombia was demonstrated 8 years later after the implementation of the program in 1992. Indeed, a significant decrease of HBsAg prevalence (60–75% reduction) was described in Amerindian children from the Amazonas and Caqueta States [21].

The prevalence of HBsAg (0.5% vs 9%) and anti-HBc (3.6% vs 30.9%) observed in children and mothers, confirm the effectiveness of the hepatitis B vaccine program. Some conditions can be related to the decline in HBV prevalence in this population. In 2001, the monovalent vaccine was replaced for the pentavalent vaccine (DPT + *Haemophilus influenzae* type b + HepB) that improved coverage with three doses of hepatitis B by increasing the vaccine acceptability for lower number of injections to which the child is subjected. Additionally, the number of women of reproductive age who are susceptible to infection with hepatitis B has decreased, because the universal vaccination began in 1994 and included children less than 5 years old, so most women under 25 years may be vaccinated. The average age of mothers is 32 years and it is likely that most of them have not been vaccinated considering that when the universal vaccination program started in Colombia most of them were more than 6 years old. The difference observed in this study between children and mothers is due mainly to increased exposure to HBV infection by mothers, and the protective effect of the vaccine in children.

The analysis of seven samples obtained from 5 mothers and 2 children successfully amplified, demonstrated that 6 of them clustered within the genotype F, which is autochthonous in America, and the most divergent HBV genotype. This genotype has been reported in America, particularly among Amerindian communities from Alaska to Argentina; indeed genotype F is prevalent in Colombia, Venezuela, Chile, Argentina, Peru, Panama, Costa Rica, El Salvador and Nicaragua [25,26,27,28,29,30].

Five of these sequences grouped with sequences from Peru, Argentina, Venezuela and Chile belonging to the subgenotype F1b. Previous studies in Amerindian communities of the Amazon Basin demonstrated the prevalence of genotype F, subgenotype F1b, in this important and vast region of South America [29,30,31,32]. F1b subgenotype has been associated with parenteral transmission and high endemicity in Amerindian populations.

The phylogenetic analysis of the HBV ORF S demonstrated a subclade of strains obtained in different communities, Puerto Esperanza (Accession number MF400838), Boyahuazu (Accession number MF400839) and San Juan de Atacuari (Accession number MF400836) belonging to subgenotype F1b. Moreover, this subclade includes the strain 520_SJ Atacuari COL AMZ previously described in Amazonas by di Filippo et al. [32]. A second subclade is shown with the sequence 180_PtoEsperanza_AM (Accession number MF400841) and the strain 862_Pto Esperanza COL AMZ previously described [32]. In addition, the sequence 048_Naranjales_AM (Accession number MF400837) was grouped with sequences from Chile, Venezuela, and Colombia in a third subclade; one of the sequences from Colombia was identified in an Amerindian from Puerto Esperanza [32] and the other one in a blood donor from the Andean region of the country [33]. The 3 subclades within the subgenotype F1b cluster could be related to independent introductions of HBV into the communities and therefore the circulation of different lineages in Amazonas.

Although, the 4 communities belong to the municipality of Puerto Nariño, they are not close to each other, in particular Puerto Esperanza to Boyahuazu, Naranjales and San Juan de Atacuari [32]. It is important to consider that the interaction among Amerindians of different communities in the Amazonas state is not frequent because the only way of transportation in the region is by the river, which is expensive for them.

Interestingly, the other sequence 020_SJ Atacuari_AM (Accession Number MF400840) within genotype F grouped with sequences of Costa Rica, El Salvador, Peru belonging to the F1a subgenotype. This subgenotype has been described in Central America where it is prevalent in Guatemala, Honduras, Nicaragua, Costa Rica and El Salvador [28,31]. F1a was described for the first time in Colombia in 2015 in an asymptomatic individual with a risk factor for HBV infection [34]. The sequence 020_SJ Atacuari AM was grouped in a subclade with a sequence from El Salvador and a sequence identified in a patient from central America receiving hospital care in Medellin [35]. Besides, this study demonstrated the circulation of two HBV subgenotypes, F1a and F1b, in a small community (159 Amerindian > 18 years old according to the data obtained from the database of the Expanded Program on Immunization) [32].

The remaining sequence 189_Tarapaca AM (Accession Number MF400842) belongs to genotype A, grouped with sequences obtained from Colombian blood donors belonging to subgenotype A2 [33]; the HBV strain was identified in a child from the Tarapaca village (North, Amazonas State). Genotype A is the second most frequent HBV genotype in Colombia as it has been described in blood donors from different cities of the Andean region of Colombia [33] and in individuals from the Pacific Coast (Choco State, West Colombia) where most of the people are Afro-descendant [33,34,36]. The introduction of genotype A into the country, as also in other Latin American countries, could be related to the influx of immigrants from Europe and Africa during the colonial period (16^th^ to 19^th^ century) [37]. The subgenotype A1 is mainly found in Africa while the subgenotype A2 in Europe. The circulation of both subgenotypes has been identified in Colombia, A1 and a recombinant strain F3/A1 in the Afro-descendant population [36], and A2 in blood donors from the Andean region [29] and as described for the first time in this study in Amerindian populations of Colombia.

The findings of this study suggest the introduction of genotype A and subgenotype F1a in communities that are geographically and culturally isolated from other Colombian populations. Indeed, the expected result was the exclusive circulation of HBV F1b in the Amerindian communities of this region as described previously [31,32], and considering the characterization of genotype F in Amerindian communities along the continent [28,31,32,33,34,36,38,39,40,41].

The present study identified two HBV escape mutants (G145R, W156*), and two potential escape mutants (L109R and G130E) in a child and a two mothers belonging to Amerindian populations of the Amazonas state.

Variations in the PreS/S ORF, mainly in the major hydrophilic region (MHR), in particular in the “a” determinant (amino acids 124 to 147 of the MHR) have been reported in samples from vaccinated children in Italy, the United States, Spain, Hungary and Taiwan [42,43,44,45,46,47,48,49,50].

The HBV escape mutants are responsible for evasion of the vaccine-induced antibodies, impairment of the immunoglobulin (HBIg) prophylaxis therapy and failure of HBsAg detection by diagnosis using commercial assays [51].

Escape mutant G145R was the first one described in a serum sample obtained from an Italian vaccinated child. In the present study, it was identified in an Amerindian mother. This mutation located in the second loop of the “a” determinant is the result of the substitution of glycine to arginine and causes a conformational change. Moreover, there are other reported escape mutants (P120T, T126A/S, and D144A/G) responsible for the evasion of vaccine-induced antibodies and therefore cause HBV infection in vaccinated individuals [15,52,53].

The reduction of HBsAg binding to monoclonal and polyclonal antibodies of HBV strains having the mutation G145R, has been demonstrated, and could due to conformational changes in the tertiary structure of the protein, thus reducing its immunogenicity. The biochemical properties of the amino acid in this domain play an important role in the recognition and binding of neutralizing antibodies to HBsAg [54,55]. Interestingly, this escape mutant is fully infectious *in vitro* as well as *in vivo*, appears stable over time and can be transmitted [14]. G145R is considered as a potential cause of a public health problem in countries with a universal vaccination program. Recently, this mutation was characterized in patients with chronic infection, cirrhosis and hepatocellular carcinoma [17].

The mutation, W156*, was identified in a sample obtained from a child having undergone the complete vaccination schedule. This stop codon is located upstream of the "a" determinant, and results in a truncated protein; this is probably why HBsAg was not detected by serological tests [56,57,58]. Interestingly, this mutation has been described in patients with end-stage liver disease but not in patients with chronic infection [17]. Similar mutations induced by antiviral treatment resulting in a truncated protein have been reported in other studies, such as W196* (31 amino acid deletion) and W172* (55 amino acid deletion) [59,60].

The stop codon was identified in a sample obtained from an Amerindian child with OBI. It is reasonable to expect escape variants associated with OBI cases, although the occult infection is more often related to suppression of HBV replication and viral gene expression by host defense mechanisms [58].

The two other mutations described in the study, L109R and G130E, have not been reported as HBV escape mutants, but they have the potential of being considered as such, since they are located in the MHR of HBsAg (amino acids 110 to 164). Unfortunately, the vaccination status of the mother from whom the HBV strain was obtained is unknown. Studies carried out in Morocco and in Vietnam reported mutations in these same positions: L109Q and G130A/N in unvaccinated patients [52,61].

The biochemical properties of amino acids in positions 109 and 130 may affect HBsAg binding to the antibodies. In the case of the L109R mutation, leucine is a nonpolar, hydrophobic amino acid, in contrast to arginine, a hydrophilic and charged residue; although this mutation is located outside of the "a" determinant but within the B-cell epitope of the MHR, it classifies as a potential escape variant. As for mutation G130E, glycine is the smallest amino acid, and changes in this position could affect the tertiary conformation of the protein and likewise its antigenicity, being replaced the negatively charged glutamic acid with a longer side chain. It is likely that differences in charge and size could modify the structure of the conformational epitope and antigen recognition by neutralizing antibodies [52,54,55]. Further studies are necessary to determine the effect on antigenicity of HBsAg with these mutations.

Taiwan is the country with the best record of escape variants. Since the beginning of the universal vaccination program, studies have been conducted every five years to evaluate the efficacy of the vaccine and frequency in the emergence of escape mutants. The studies showed that mutation G145R is the most prevalent, 20 years after the introduction of the vaccine. Also there has been an increase in the frequency of escape mutants, from 7.8% in 1984, before the introduction of the vaccine, to 22.6% in 2004 [47].

A study carried out in Hungary, a country with moderate endemicity for HBV infection identified HBV escape mutants in 28 children vaccinated and positive for the serological markers HBsAg and/or anti-HBc, and in 40 women positive for anti-HBc. The study identified the P120T and G130S mutations as the most frequent in both groups. The frequency of escape mutants was 33.3% (4/12) in children and 47.3% (9/19) in women [49].

A study conducted in the USA in HBsAg+ vaccinated children, born to HBsAg+ mothers showed that 23.4% (22/94) of the children presented escape mutants [50]. Although USA is a country with low prevalence of HBV infection, this frequency of escape mutants is similar to that found in the present study (28.5%).

In Latin America, the escape mutant D144A/G was characterized in a vaccinated child and potential escape mutants were identified in HBsAg+/anti-HBs+ patients in Argentina [16].

The emergence and stabilization of escape mutants to different viral vaccines worldwide are events that behave like a Darwinian evolution process. However, these mutations occur spontaneously and randomly under the pressure of the immune response in vaccinated or infected individuals and also after anti-HBV immunoglobulin therapy [54,62].

Concerning the HBV vaccine, the selection by immunization in endemic populations for HBV infection has not favored the emergence of escape mutants, probably because these mutations confer a selective disadvantage in competition with the wild-type virus [51]. In contrast, the study of Hsu *et al*. demonstrated that infants born to HBsAg+ mothers are a population in which the spread of escape mutants more readily occurs [48].

In regions with high prevalence of infection, where these variants have been found, the recommendation is to implement new vaccines containing the common escape mutants, mainly G145R [46,48].

Although, there are no studies that assess the epidemiological impact and biological and clinical significance of escape mutants, it is important to detect the circulation of these variants, implementing systematic surveillance and monitoring the mutants to keep control of their spread in high prevalence regions.

In conclusion, we reported HBV Genotype F (85.7%) and Genotype A (14.2%) circulating in the Amazonas state, Colombia. To the best of our knowledge, this is the first characterization of subgenotype F1a and Genotype A in Amerindian communities in Colombia.

Additionally, the first description of HBV escape mutants in Colombia is an interesting result. However, additional studies are necessary to elucidate the frequency and the epidemiological impact of the escape mutants in this country.

## Supporting information

S1 Database Database of a cross-sectional survey designed to evaluate the prevalence of HBV and vaccine coverage in children population of rural communities in the Amazonas State, Colombia. Database variables of the study carried out from June 2010 and June 2011 on 1275 children belonging to 37 amerindian communities settled on the banks of the Putumayo and Amazonas rivers and theirs tributaries in the Amazonas State, Southern Colombia.
(XLSX)

S2 Database Database of a cross-sectional survey designed to evaluate the prevalence of HBV in mothers of rural communities in the Amazonas State, Colombia. Database variables of the study carried out from June 2010 and June 2011 on 572 mothers belonging to 37 amerindian communities setllted on the banks of the Putumayo and Amazonas rivers and theirs tributaries in the Amazonas State, Southern Colombia.
(XLSX)
